# Effects of dietary supplementation with grape seed powders on growth performance and muscle nutrition of grass carp (*Ctenopharyngodon idella*) by gut microbiota mediation

**DOI:** 10.3389/fphys.2025.1683389

**Published:** 2025-12-12

**Authors:** Shuaipeng Ma, Qing Liu, Qianqian Chen, Songqing Nie, Yulin Zhang, Gang Wu, Xuesong Wang

**Affiliations:** 1 Guangdong Eco-engineering Polytechnic, Guangzhou, China; 2 Guangzhou Experimental Station of Chinese Academy of Tropical Agricultural Sciences, Guangzhou, China; 3 Key Laboratory of Chinese Medicinal Resourse from Lingnan, Ministry of Education, School of Pharmace Sciences, Guangzhou University of Chinese Medicine, Guangzhou, China

**Keywords:** grape seed powders, procyanidins, aquaculture, grass carp, growth performance

## Abstract

**Objective:**

To investigate whether grape seed powders (GSP) contribute to growth-promoting in aquaculture, a comprehensive analysis of the effects of GSP on grass carp (*Ctenopharyngodon idella*) was performed.

**Methods:**

Six hundred grass carp were divided into four groups, including the control and the low (100 mg/kg), middle (500 mg/kg), and high (1000 mg/kg) GSP-supplemented groups. The material composition, growth parameters, physiological/biochemical indexes, and muscle nutrition were analyzed.

**Results:**

After feeding of 60 days, the weight gain rate, specific growth rate, and condition factor of grass carp significantly increased with GSP supplementation at low and middle levels in comparison to the blank control (*P* < 0.05). Additionally, the catalase activities in the gill and gut significantly elevated as dietary supplementation with GSP at the low and middle levels in comparison to the blank control (*P* < 0.05), while the interleukins’ (IL-1*β*, IL-4, IL-6, IL-10, and IL-12) contents in the spleen significantly decreased (*P* < 0.05), suggesting an enhancement in antioxidant capacity activities and a reduction in inflammation levels after supplemental feeding with GSP. The total amino acids and total fatty acids in the blank control were equivalent to these in the low GSP-supplemented group, which were inconsistent with the upregulation of total amino acids and downregulation of total fatty acids in the middle and high GSP-supplemented groups. Furthermore, pathogenic bacteria in the gut (e.g., *Enterobacter hormaechei* and *Enterobacter cloacae*) were effectively inhibited in the GSP-supplemented groups, with significant correlations to the increased amino acid (Pro) and the decreased fatty acids (e.g., C16:1n7, C18:1n9c, C20:2n6, and C22:6n3) (*P* < 0.05).

**Conclusion:**

Based on the results, we confirmed that dietary supplementation with procyanidin-rich GSP at a relative low level (100 mg/kg) was beneficial for the healthy aquaculture of grass carp.

## 1 Introduction

The grape (*Vitis vinifera* L.), widely acknowledged as a significant economic fruit, is cultivated extensively across the globe (Wang et al., 2024). In addition to its fruit profile, grape exhibits diverse therapeutic properties and has served as a traditional Chinese medicinal material for thousands of years (Quero et al., 2021). Evidence demonstrates that grape possesses numerous pharmacological and clinical effects in combating DNA oxidative damage, cardiovascular diseases, diabetes, hepatopathy, neuropathy, and other health conditions, attributed to its bioactive constituents (Coelho et al., 2023; Nassiri-Asl and Hosseinzadeh, 2016; Zhao et al., 2020), which include flavonoids, polyphenols, anthocyanins, and stilbene derivatives. Recently, these bioactive components have commonly been identified in the fruits, seeds, leaves, stems, and pomaces of the grape (Toaldo et al., 2015). Among these, grape seeds have garnered considerable attention due to their substantial value in the expansive health industry, such as cardioprotective, neuroprotective, hepatoprotective, antiviral, antioxidative, anti-inflammatory, anti-aging properties, and enhancement of gastrointestinal motility (Choi et al., 2022; Jebari et al., 2022; Talebi et al., 2023).

It is well established that the utilization of grape seed (including flour, extract, and pomace) as a feed additive for promoting growth and preventing diseases has been implemented within the poultry and livestock sectors (Hassan et al., 2019), encompassing species such as chickens, hens, geese, ducks, weaned piglets, goats, and calves (Abu Hafsa and Hassan, 2022; Ao and Kim, 2020; Deng et al., 2023; Guo et al., 2020; Liu et al., 2018; Ma et al., 2023; Yang et al., 2014). Unfortunately, in contrast to terrestrial animals, the relevant feeding validation concerning aquatic animals remains limited. Furthermore, procyanidins, which fall under the category of polyphenols, are the primary essential components of grape seed, and the procyanidin-rich grape seed extract manifests commendable to exceptional health-protecting effects through the modulation of associated signaling pathways, which contributes growth-promoting in animal farming (Baron et al., 2024; Ricketts and Ferguson, 2018). Recently, increasing number of literatures had reported the application of polyphenols and polyphenol-rich additives in aquaculture, which could apparently enhance the health status and growth of fishes due to antioxidant capacity elevation (Yang et al., 2023; Mohammadi et al., 2021), immunity amelioration (Ahmadifar et al., 2020), and gastrointestinal toxicity mitigation (El-Fahla et al., 2025), and disease resistance increase (Mehrinakhi et al., 2020). Consequently, grape seed with a high procyanidin content may serve as an ideal substance for use as a feed additive in animal husbandry, particularly in the realm of aquaculture.

Grass carp (*Ctenopharyngodon idella*), recognized as the principal domestic carp in China, constitutes the most significant freshwater fish species in the nation (Lu et al., 2020a). As one of the four major Chinese carps, grass carp grow fast with a high meat yield. Meanwhile, the muscle of grass carp is rich in unsaturated fatty acid and tastes delicious (Yu et al., 2014). However, due to anthropogenic activities, including water pollution, overfishing, and vessel navigation, the population of grass carp in the primary rivers of China, such as the Yangtze River and Pearl River, has experienced a notable decline over the past 4 decades, comparing data from the 1980s to that of the 2020s (Mai et al., 2023). Furthermore, global production of grass carp has reached approximately 6.07 million tons, which represents a substantial portion of total global fish production (Yu et al., 2020). Consequently, there is an urgent need for sustainable aquaculture practices for grass carp to satisfy both domestic and international demand. To maintain the normal production of grass carp, antibiotics were commonly used; however, the Chinese government has prohibited the application of antibiotics since 1 July 2020. Therefore, the alternative to antibiotics is emergently needed. Currently, Chinese medicinal materials are being utilized as alternatives to antibiotics, and the incorporation of plant-based ingredients into diets has significantly enhanced the disease resistance and growth performance of fish (Hemre et al., 2016). In light of the above-mention aspects, the effects of grape seed on the growth performance and muscle quality of grass carp had aroused our interest, which could facilitate the healthful aquaculture of grass carp.

In the present work, we aimed to evaluate the ability of grape seed powders (GSP) to elevate growth performance in aquaculture by using grass carp as an experimental animal. Material composition analysis, growth parameters measurements, physiological/biochemical indexes determination, and muscle nutrition detection were conducted in the laboratory. Meanwhile, histology inspection and gut microbiome analysis were performed at the same time. We hypothesized that feeding inclusion GSP was growth-promoting in the healthful aquaculture of grass carp. Our study is conducive to broaden the application prospect of grape seed for serving as feeding additive in the aquaculture industry.

## 2 Materials and methods

### 2.1 Preparation of GSP

The grape seeds were acquired from the Kyoho variety, provided by Guangdong Eco-engineering Polytechnic (Guangzhou, China). After being thoroughly washed with deionized water for three times, the grape seeds were dried at 40 °C for 7 days in an oven. Subsequently, the dried grape seeds were milled using a cyclone mill, and the resultant grape seed powder was collected.

### 2.2 Material composition analysis

The assessment of bioactive constituents (including procyanidin, polyphenols, flavonoids, and polysaccharides), pesticide residues (namely dichlorodiphenyl trichloroethane (DDT), hexachlorocyclohexane (HCH), and quintozene (PCNB)), heavy metals (such as lead (Pb), cadmium (Cd), mercury (Hg), and arsenic (As)), and microbial content (including *Salmonella*, *Staphylococcus aureus*, aerobic bacterial counts, molds and yeasts, and coliforms) within the prepared GSP was conducted in accordance with the national food safety standards of China. These standards include GB/T 22244-2008, GB/T 8313-2018, GB/T 40632-2021, GB/T 5009.19-2003, GB/T 5009.136-2003, NHC (2016a), NHC (2016b), NHC (2016c), NHC (2016d), NHC (2016e), and NHC (2016f), respectively. For procyanidin detection, 1 g GSP was extracted with 20 mL methanol (ultrasonic, 400 W, 25 °C, 30 min). Then, the extraction liquid was centrifuged (4,000 r/min, 10 min). These extraction processes were repeated twice. After dilution to 50 mL and filtration with a 0.45 μm pore, the supernatant was analyzed using HPLC system (C18 column; methanol-0.1% formic acid, 60:40; 1 mL/min; 525 nm). Quantification was performed based on procyanidin standard curve (10–150 μg/mL). For polyphenols and flavonoids measurements, 0.5 g GSP was refluxed with 10 mL 70% ethanol (80 °C, 2 h). After centrifugation, the supernatant was diluted to 25 mL. Of these, 1 mL supernatant was added with 5 mL Folin-Ciocalteu reagent and 4 mL 7.5% Na_2_CO_3_, and then incubated in dark for 1 h. The polyphenols content was computed based on the gallic acid standard curve. Simultaneously, 2 mL supernatant was added with 0.5 mL 5% NaNO_2_ and 0.5 mL 10% Al(NO_3_)_3_. After standing for 6 min, 10 mL 4% NaOH was added and the mixture was diluted to 25 mL subsequently. The flavonoids was calculated based on rutin standard curve. For polysaccharides determination, 2 g GSP was refluxed with 80 mL water (95 °C, 2 h). After precipitation with 70% ethanol (4 °C, overnight), the dry precipitate (crude polysaccharides) was obtained. Subsequently, the crude polysaccharides was hydrolyzed with 5 mL 1 mol/L HCl (100 °C, 2 h). About 1 mL hydrolysate was mixed with 1 mL 5% phenol and 5 mL H_2_SO_4_. The polysaccharides content was assessed at 490 nm based on glucose standard curve (0–60 μg/mL). For heavy metals detection, 0.5 g GSP was digested with HNO_3_-H_2_O_2_ via microwave program (180 °C, 15 min). Subsequently, the digestion solution was diluted to 25 mL and filtered with a 0.45 μm pore. The heavy metals contents were analyzed using ICP-MS method. For pesticide residues analysis, 2 g GSP was extracted with 10 mL acetonitrile (vortex 1 min) and dried at 40 °C under N_2_ flowing. The pesticide residues concentrations were evaluated using GC/LC-MS method. For microorganisms observation, 25 g GSP was added into 225 mL saline. After homogenization and ten-fold dilution, the suspension was incubation at different conditions. The aerobic bacterial counts was counted after incubation at 36 °C for 48 h; the molds and yeasts was counted after incubation at 28 °C for 5 days, and the pathogens was determined after incubation in buffered peptone water.

### 2.3 Animals and feeding experiment

Due to the allometric growth factor b of grass carp reached to 3.09 (https://www.fishbase.org/summary/SpeciesSummary.php?ID=79&AT=grass+carp), the juvenile grass carp, with an average body weight of 27.1 g and an average body length of 12.1 cm, were procured from Fulong Aquaculture Company (Guangzhou, China). All specimens were acclimated in tanks (48.7 × 34.3 × 25.8 cm (50 L) for 1 w without feeding. During acclimatization, the dissolved oxygen (DO) concentration was 8.2 ± 0.3 mg/L, the pH level was 7.4 ± 0.2, the water hardness (expressed as CaCO_3_) was 34.8 ± 0.5 mg/L, the salinity was 0.10‰ ± 0.03‰, the nitrate nitrogen (NO_3_-N) concentration was 1.4 ± 0.1 mg/L, and the water temperature was 25 °C ± 0.5 °C (Supplementary Table S1). No mortality was determined among the specimens. After acclimation, a total of 600 fish were randomly allocated into four groups with different treatments: the Control (CK) group, which received basal feed pellets (BFP); the Treatment Low (TL) group, fed BFP with 100 mg/kg GSP; the Treatment Medium (TM) group, fed BFP with 500 mg/kg GSP; and the Treatment High (TH) group, fed BFP with 1,000 mg/kg GSP. Consequently, 150 fish were assigned to each treatment, with 50 fish per tank (48.7 × 34.3 × 25.8 cm, 50 L) serving as the experimental unit. The BFP, referred to as 1,038 original pond particles, was acquired from Tongwei Group in Chengdu, China, and their composition is detailed in Supplementary Table S2. To prepare the compound feed pellets, BFP and GSP underwent filtration through 200 mesh stainless steel and were thoroughly homogenized. After homogenization, sterile water was introduced, with a dietary to water ratio established at 4:1 (weight/volume). Subsequently, the moist compound feed pellets were re-pelletized using a stainless steel feed pellet machine and then dried at 56 °C for 1 h, so that the moisture content of the compound feed pellets were below 10%. The resultant compound feed pellets were utilized in feeding trials without other specified. Throughout the feeding experiments, the rearing conditions kept in line with the acclimatization period, and the feeding duration was 60 days, in accordance with the guidelines for assessing the effects of feed additives/supplants on physiological aspects in fish. When feeding finished, the final body lengths and weights of 30 fishes were measured randomly (10 per tank). The fishes were euthanized utilizing Sigma-Aldrich® tricaine methanesulfonate (MS-222, 5 ppm) by using NaHCO_3_ solution as buffer. All experiments were conducted in agreement with the approved ethical guidelines (LAEC-PRFRI-2023-06-07).

### 2.4 Sample collection

Three tissues (gill, gut, and muscle) were individually collected from fish specimens (n = 10). Fifty percent of the collected tissues were preserved in 4% paraformaldehyde for histological examination, while the remaining fifty percent were stored at −80 °C for the assessment of physiological and biochemical indices, as well as for the evaluation of muscle nutritional content. In addition, the gut contents from the fish specimens (n = 3, the mixed gut contents of 10 individuals in each tank) were collected and preserved for subsequent gut microbiome sequencing and analysis. Likewise, the gut contents were stored at −80 °C.

### 2.5 Growth parameter measurements

The growth parameters, which include weight gain rate (WGR, %), specific growth rate (SGR, %/d), condition factor (CF, %), feeding coefficient (FC, %), hepatosomatic index (HSI, %), and viscerosomatic index (VSI, %), were meticulously measured and computed in accordance with established methodologies formulas (Wang et al., 2025):

$$WGR=\left(final\;body\;weight-initial\;body\;weight\right)\;initialbody\;weight\times 100;$$

$$SGR=\left(\ln\left(final\;body\;weight\right)-\ln\left(initial\;body\;weight\right)\right)÷feeding\;days\times 100;$$

$$CF=final\;body\;weight{/\left(final\;body\;length\right)}^{3}\times 100;$$

$$FC=feed\;consumption/\left(final\;body\;weight-initial\;body\;weight\right)\times 100;$$

HSI=final liver weight/final body weight×100;

VSI=final visceral weight/final body weight×100;

### 2.6 Histology inspection

The selected tissues (gill, gut, and muscle) were fixed using a 4% (v/v) paraformaldehyde solution (n = 10). Subsequently, the paraformaldehyde-fixed tissues were dehydrated with graded ethanol solutions, cleared with Sigma-Aldrich® xylene, embedded in Sigma-Aldrich® paraffin, and thereafter sliced into sections of 5 μm thickness utilizing a Leica® microtome. The obtained sections were rehydrated and stained with Sigma-Aldrich® hematoxylin and eosin (H&E) twice and then mounted utilizing a neutral gum medium. Finally, the prepared sections were examined and imaged using an ECHO REVOLVE® microscope. The ratios of secondary lamellae length to width, villi length, and muscle fiber diameters pertaining to the gill, gut, and muscle were determined using ImageJ software.

### 2.7 Physiological/biochemical indexes determination

The selected tissues (gill, gut, and muscle) were underwent comprehensive physiological and biochemical analyses (n = 10). The tissues were homogenized in ice-cold double distilled water. The assays for superoxide dismutase (SOD), catalase (CAT), malondialdehyde (MDA), glutathione peroxidase (GPx), glutathione S-transferase (GST), and glutathione (GSH) were conducted using Beijing Solarbio® colorimetric test kits (Science and Technology Co., Ltd., Beijing, China), in strict accordance with the manufacturer’s protocols. Additionally, the levels of immunoglobulins (IgA, IgG, and IgM) and interleukins (IL-1*β*, IL-2, IL-4, IL-6, IL-10, and IL-12) were assessed employing Shanghai Enzyme-linked® colorimetric kits, also following the manufacturer’s instructions.

### 2.8 Muscle nutrition detection

The composition of muscle amino acids and fatty acids was analyzed in accordance with the national food safety standards of China, specifically NHC (2016g) and NHC (2016h), respectively. For amino acid assessment (n = 3), the muscle samples were subjected to acid hydrolysis utilizing 6 M HCl and subsequently freeze-dried via a vacuum freeze dryer under nitrogen conditions. Following hydrolysis at 110 °C for 22 h, the resultant hydrolysate was dried at 40 °C using a vacuum, and then mixed with sodium citrate buffer (1 mL) at pH 2.2. The mixture was filtered through a filter with a pore size of 0.22 μm, and the obtained supernatant was transferred to autosampler vials for instrumental analysis. During this analysis, a Thermo Accucore XL C-18 column (2.0 × 100 mm, 4 μm) was employed for separation, and the samples were underwent further analysis using an HPLC system (LC-20D, Shimadzu, Kyoto, Japan). The mobile phase was composed of component A (methanol), and the total run time was 10 min with a flow rate of 1.2 mL/min. The column’s temperature was maintained at 30 °C, and an injection volume was 10 μL. For fatty acid assessment (n = 3), gas chromatography (GC) analysis was conducted using a GC-2010 plus (Shimadzu, Kyoto, Japan) equipped with an HP-88 column (100 m × 0.25 mm × 0.2 μm, Agilent, California, United States). Detection was performed using a flame ionization detector.

### 2.9 Muscle safety inspection

To ensure the safety of muscles for consumption, the presence of pesticide residues (DDT, HCH, and PCNB), heavy metals (Pb, Cd, Hg, and As), and foodborne pathogenic microorganisms (*Salmonella* and *Escherichia coli*) was monitored in accordance with the standards established in GB/T 5009.19-2003, GB/T 5009.136-2003, NHC (2016a), NHC (2016b), and NHC (2016c), respectively. The experimental procedures and measurement techniques were in consistent with these in “2.2. Material composition analysis.”

### 2.10 Gut microbiome sequencing

The gut microbiome analysis was performed at Beijing Biomarker Technologies Co., LTD (Beijing, China). DNA was extracted from each sample (n = 3 per group, the mixed gut contents of 10 individuals in each tank) utilizing a TGuide S96 Magnetic Soil/Stool DNA Kit (Tiangen Biotech (Beijing) Co., Ltd., Beijing, China) according to manufacturer instructions, and the concentration of the DNA was measured using a Qubit dsDNA HS Assay Kit and Qubit 4.0 Fluorometer (Invitrogen, Thermo Fisher Scientific, Oregon, United States). The 16 S rRNA genes of the V3–V4 regions were amplified employing specific primers (forward primer: 5′- ACTCCTACGGGAGGCAGCA- 3′, reverse primer: 5′- GGACTACHVGGGTWTCTAAT- 3′) that included a 12-bp barcode. PCR amplification was executed in a 25-μL reaction system, which consisted of 5 μL of 5 × reaction buffer, 5 μL of 5 × GC buffer, 2 μL of 2.5 mM dNTP, 1 μL of 10 μM forward primer, 1 μL of 10 μM reverse primer, 2 μL of 20 ng/μL DNA template, 0.25 μL of Q5® High- Fidelity DNA polymerase (NEB™), and 8.75 μL of ddH_2_O. The PCR reactions were amplified utilizing an ABI™ 2720 PCR instrument following the thermocycling protocol: initial denaturation at 98 °C for 2 min; 30 cycles of denaturation at 98 °C for 15 s, annealing at 55 °C for 30 s, and extension at 72 °C for 30 s; concluding with a final extension at 72 °C for 5 min. The PCR products were amalgamated and purified using Vazyme™ VAHTSTM DNA clean beads. Sequencing libraries were generated using an Illumina® TruSeq Nano DNA LT Library Prep kit, subsequently quality-assessed on an Agilent™ Bioanalyzer, and ultimately sequenced on an Illumina® Miseq 2500 platform, resulting in 250-bp paired-end reads.

### 2.11 Bioinformatic analysis

Following quality filtering, denoising, and chimera removal, the paired-end clean amplicon sequence variants (ASVs) was generated using DADA2, with counts less than two among the tested groups. At a confidence threshold of 70%, the taxonomic annotation of ASVs was carried out based on the Naive Bayes classifier within QIIME2, by matching with the SILVA database (release 138.1). Alpha diversity (Shannon and Simpson indexes) and beta diversity (based on principal coordinate analysis (PCoA)) were performed to assess within-group diversity and the degree of similarity between groups. Meanwhile, to identify the taxonomic differences between groups, Linear Discriminant Analysis Effect Size (LEfSe) was conducted, with a logarithmic LDA score threshold of 4.0 for discriminative features. Additionally, the potential keystone species was identified using Extreme Gradient Boosting (XGBoost) machine learning. During analysis, only features with an importance score above than 0.1 were visualized. Furthermore, the functional composition of gut microbiota was predicted using PICRUSt2. The analysis, including alpha diversity, beta diversity, and machine learning, were carried out using the EasyMultiProfiler package within RStudio software.

### 2.12 Antibacterial validation

The antibacterial efficacy of the prepared GSP against *Enterobacter cloacae* and *E*. *hormaechei* was assessed in accordance with GB 15979-2002, incorporating minor modifications. Two bacterial suspensions (1 × 10^3^ and 1 × 10^4^ CFU/mL for each species) were prepared, and the concentrations of GSP were set at 5.0 and 25.0 mg/L, respectively. Subsequently, 100 μL of the prepared bacterial suspensions and 0.5 mL of the prepared GSP solution were introduced into a petri dish containing 15 mL of Mueller Hinton/potato dextrose agar medium. Following this, the solidified petri dish was incubated at 35 °C for 48 h. Pure DMSO was served as the negative control, while a bacterial suspension was acted as the blank control. Ultimately, the antibacterial activity of the prepared GSP against the selected gut bacteria was quantified based on the follow formula: I = (A−B)/A × 100%, where “I” was inhibition rate (%), “A” was the average colony count of control, and “B” was the average colony count of treatment.

### 2.13 Statistical analysis

All data, including growth parameters, histological indices, physiological and biochemical indices, concentrations of amino acids and fatty acids, alpha diversity in gut microbiota, and inhibition rates of procyanidin-rich GSP, were represented as mean ± standard deviation (SD). Prior to the statistical analysis, the normality of the data was assessed using the Shapiro-Wilk test. In cases where the data were normally distributed, a parametric one-way ANOVA accompanied by Fisher’s Least Significant Difference (LSD) test was employed to evaluate significant differences among groups. Conversely, when the data were not normally distributed, a nonparametric Kruskal–Wallis test followed by Dunn’s test was utilized to determine significant differences among groups. All statistical tests were listed in Figure legends. Additionally, the Spearman’s correlation analysis was performed to examine the relationships between the bacterial taxa, the predicted functional profiles, the muscle enzymatic/nutritional, and the texture parameters based on a threshold of Spearman’s *r* > 0.3 and *P* < 0.05. All statistical analyses and plot generation (including histograms and scatter plots) were conducted using GraphPad Prism 10 software (version 10.2.1).

## 3 Results

### 3.1 Material composition of the prepared GSP

The material composition of the prepared GSP is detailed in Supplementary Table S3. The concentrations of bioactive constituents within the GSP, including procyanidine, polyphenol, flavonoid, and polysaccharide, were 10.40, 25.80, 3.56, and 0.39 g/100 g, respectively. Additionally, the levels of heavy metals, pesticide residues, and microbial content in the GSP were evaluated, all of which conformed to the national food safety standards of China.

### 3.2 Growth performance of grass carp

**FIGURE 1:**
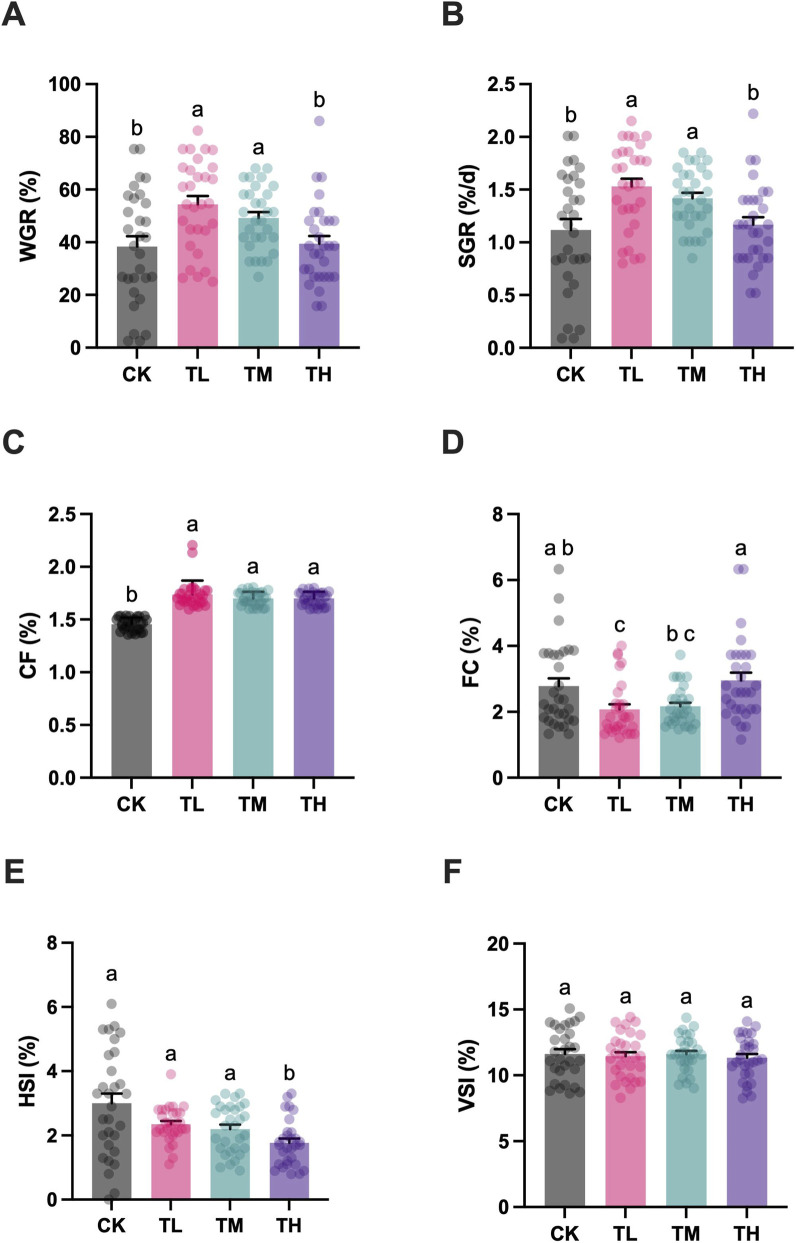
Growth performance of grass carp after supplemental feeding with procyanidin-rich GSP (n = 30). **(A)**, weight growth rate (WGR); **(B)**, specific growth rate (SGR); **(C)**, conditional factor (CF); **(D)**, feeding coefficient (FC); **(E)**, hepatosomatic index (HSI); and **(F)**, viscerosomatic index (VSI). Lowercase letters indicate the significant difference between the different groups (One-way ANOVA for WGR and SGR; Kruskal Wallis test for CF, FC, HSI and VSI; *P* < 0.05). The type of error bars is standard deviation (SD).

Significant upregulation in WGR and SGR was determined between the blank control (CK group) and the low and middle GSP-supplemented groups (TL and TM group) (*P* < 0.05) (Figures 1A,B). Meanwhile, the WGR and SGR were the highest in TL group, exhibiting a significant difference compared to TH group (*P* < 0.05). Similarly, the CF in the blank control (CK group) was significantly lower than that in the GSP-supplemented groups (TL, TM, and TH groups) (*P* < 0.05), with the highest CF in TL group (Figure 1C). Conversely, the FC in TL group significantly decreased when compared to both the blank control (CK group) and the high GSP-supplemented group (TH group) (*P* < 0.05) (Figure 1D). Therefore, dietary addition of GSP at a relative low level (100 mg/kg) manifested a growth-promoting effect, accompanied by an enhanced feed conversion ratio. Interestingly, the HSI in the high GSP-supplemented group (TH group) was significantly lower than that in the blank control (CK group) (*P* < 0.05) (Figure 1E). Moreover, no significant difference in the VSI was determined between groups (*P* > 0.05) (Figure 1F).

### 3.3 Histological changes of grass carp

**FIGURE 2:**
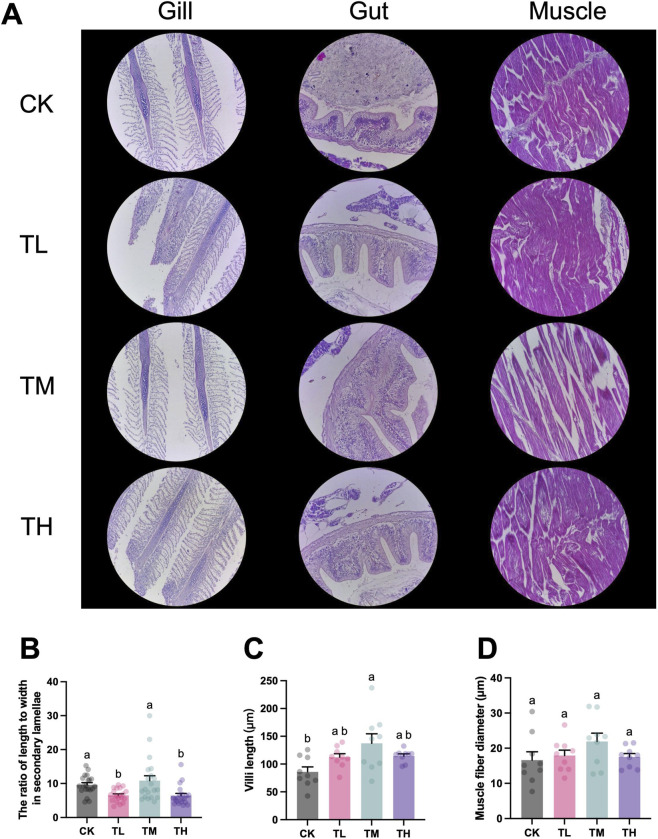
Histological changes in grass carp after supplemental feeding with procyanidin-rich GSP (n = 10). **(A)**, Representative images of the gill (200×), gut (200×), and muscle (200×) in grass carp among the four tested groups; **(B)**, the ratio of length to width of the secondary lamellae in the gill; **(C)**, villi length in the gut; and **(D)**, muscle fiber diameters. Lowercase letters indicate the significant difference between the different groups (One-way ANOVA for villi length and muscle fiber diameter; Kruskal Wallis test for the ratio of length to width in secondary lamellae; *P* < 0.05). The type of error bars is standard deviation (SD).

The ratio of length to width in the secondary lamellae of the gill in the blank control (CK group) was significantly higher than that in the low and high GSP-supplemented groups (TL and TH groups) (*P* < 0.05) (Figures 2A,B). Notably, the villi length increased in all of the GSP-supplemented groups (TL, TM, and TH groups) when compared to the blank control (CK group), with a statistically significant difference between the CK and TM groups (*P* < 0.05) (Figures 2A,C). However, no statistically significant difference was determined between the blank control (CK group) and the GSP-supplemented groups (TL, TM, and TH groups) (*P* > 0.05) (Figures 2A,D).

### 3.4 Antioxidant and detoxification indexes of grass carp

**FIGURE 3:**
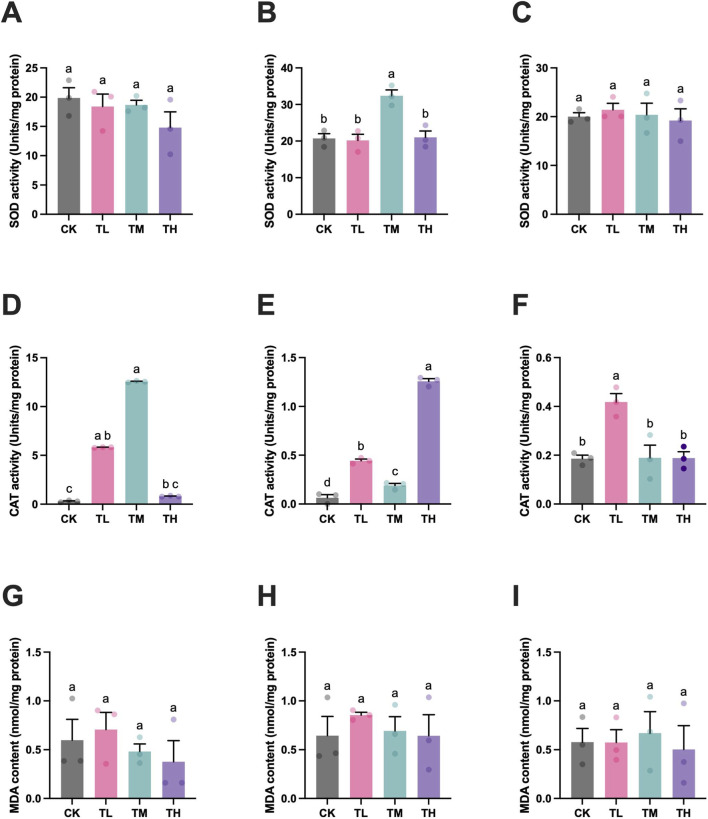
The activities/content of antioxidative enzymes/component in tissues of grass carp after supplemental feeding with procyanidin-rich GSP (n = 10). **(A,D,G)**, gill; **(B,E,H)**, gut; and **(C,F,I)**, muscle. Lowercase letters indicate the significant difference between the different groups (One-way ANOVA for SOD and MDA; Kruskal Wallis test for CAT; *P* < 0.05). The type of error bars is standard deviation (SD).

No consistent changes in SOD or CAT activities were observed among the three tested tissues (gill, gut, and muscle) between groups. Specifically, the SOD activity in the gut was significantly elevated in the TM group in comparison to the CK group, while the CAT activities in the gill, gut, and muscle exhibited significant enhancement in TL group when compared to the CK group (Figures 3A–F). Moreover, the CAT activities in the gill and gut were significantly higher in the middle and high GSP-supplemented groups (TM and TH groups) than these in the CK group (*P* < 0.05), apart from the CAT activity in gill of TH group. Additionally, the MDA contents in the gill, gut, and muscle showed no significant differences between groups (*P* > 0.05) (Figures 3G–I). Consequently, the increase in SOD and CAT activities across the three tested tissues implied an enhanced antioxidant capacity of grass carp as daily addition of GSP.

Meanwhile, the GST activity in the muscle significantly increased with daily supplements of GSP (TL, TM, and TH groups) when compared to the blank control (CK group) (*P* < 0.05). Conversely, GPx activity in the gill and gut significantly decreased with dietary addition of GSP (TL, TM, and TH groups) in comparison to CK group (*P* < 0.05), with the exception of GPx activity in the gill of TM group (Supplementary Figures S1A–F). Furthermore, the GSH content in the gut demonstrated a significant increase in the middle GSP-supplemented group (TM group) in comparison to the blank control (CK group) (*P* < 0.05), while the GSH content in the muscle exhibited significant reduction in all GSP-supplemented groups (TL, TM, and TH groups) when compared to CK group (*P* < 0.05) (Supplementary Figures S1G–I). Additionally, the GSH content in the gill revealed no significant difference between groups. Therefore, an enhanced detoxification capacity in the muscle was noticed following supplemental feeding with GSP.

### 3.5 Immunologic indexes of grass carp

**FIGURE 4:**
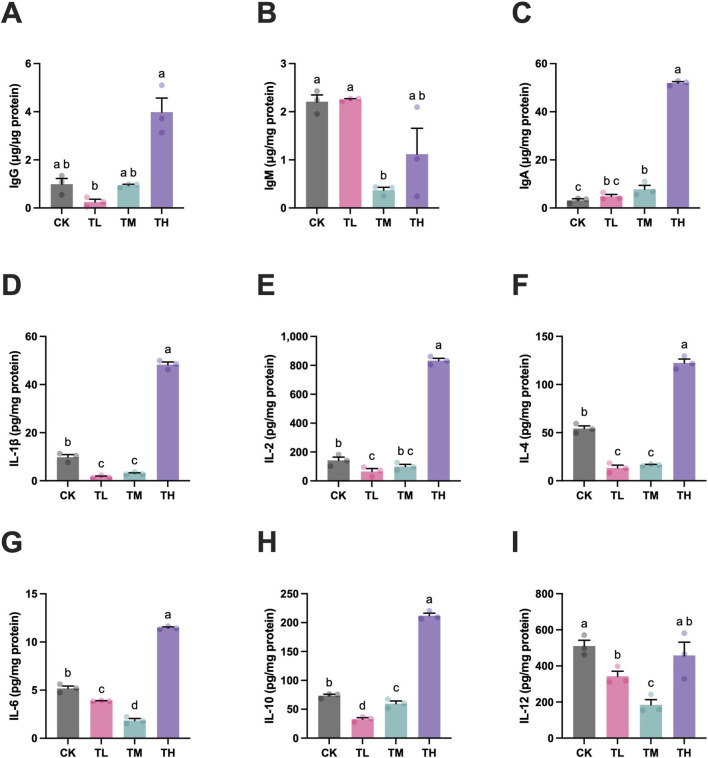
The contents of immunologic indexes in spleen of grass carp after supplemental feeding with procyanidin-rich GSP (n = 10). **(A)**, IgG; **(B)**, IgM; **(C)**, IgA; **(D)**, IL-1*β*; **(E)**, IL-2; **(F)**, IL-4; **(G)**, IL-6; **(H)**, IL-10; and **(I)**, IL-12. Lowercase letters indicate the significant difference between the different groups (One-way ANOVA for IgA, IL1β, IL2, IL4, IL6, IL10, IL12; Kruskal Wallis test for IgG an IgM; *P* < 0.05). The type of error bars is standard deviation (SD).

The levels of IgG and IgA in the TL group were significantly lower than those in the TH group (*P* < 0.05) (Figures 4A,C). Meanwhile, the IgA level significantly upregulated in both of the TM and TH groups when compared to the CK group (*P* < 0.05), while the IgM level significantly downregulated in the TM group in comparison to the CK group (*P* < 0.05) (Figures 4B,C). Simultaneously, significant reduction in the levels of IL-1*β*, IL-2, IL-4, IL-6, IL-10, and IL-12 were observed in the TL and TM groups if comparison with the CK group (*P* < 0.05). In contrast, significant elevation were acknowledged with GSP supplementation at the high level (TH group) (*P* < 0.05), except IL-2 in the TM group and IL-12 in the TH group (Figures 4D–I). These findings suggested that daily supplementation with GSP at low and middle levels is beneficial for inflammation relief, whereas daily supplementation with GSP at a high level may trigger an immune response and inflammatory reaction.

### 3.6 Muscle amino acids and fatty acids of grass carp

Five amino acids- Aspartic acid (Asp), Glutamic acid (Glu), Alanine (Ala), Lysine (Lys), and Leucine (Leu)- were predominant amino acids due to their contents exceeding the threshold of 1.00 g/100 g (Table 1). Of these, Glu was the most abundant. Proline (Pro), the sole amino acid demonstrating significant difference between groups (*P* < 0.05), showed an increase with daily supplement of GSP (TL, TM, and TH groups) when compared to the CK group. Consequently, the total amino acids (TAAs) in the GSP-supplemented groups (TL, TM, and TH groups) were elevated in comparison to the blank control (CK group) and significant differences were observed between TM/TH and CK/TL groups (*P* < 0.05). This observation suggests that dietary supplementation with GSP affected muscle amino acid composition through the elevation of Pro level.

**TABLE 1:** The muscle amino acids composition of grass carp after supplemental feeding with procyanidin-rich GSP (n = 3).

Item	Amino acid (g/100 g FW)	CK	TL	TM	TH
DAA	Asp	1.46 ± 0.07	1.51 ± 0.10	1.55 ± 0.07	1.54 ± 0.03
	Glu	2.53 ± 0.13	2.59 ± 0.17	2.63 ± 0.12	2.58 ± 0.13
	Phe	0.62 ± 0.09	0.64 ± 0.10	0.66 ± 0.02	0.63 ± 0.02
	Gly	0.67 ± 0.04	0.73 ± 0.05	0.72 ± 0.03	0.74 ± 0.01
	Tyr	0.58 ± 0.06	0.56 ± 0.06	0.61 ± 0.04	0.60 ± 0.02
	Ala	1.02 ± 0.04	1.06 ± 0.08	1.06 ± 0.03	1.06 ± 0.01
SAA	Lys	1.32 ± 0.15	1.32 ± 0.17	1.47 ± 0.06	1.39 ± 0.02
	Pro	0.48 ± 0.10^b^	0.76 ± 0.13^a^	0.84 ± 0.19^a^	0.95 ± 0.19^a^
	Ser	0.56 ± 0.04	0.54 ± 0.09	0.61 ± 0.02	0.59 ± 0.01
	Thr	0.62 ± 0.04	0.59 ± 0.10	0.66 ± 0.02	0.66 ± 0.01
BAA	Val	0.70 ± 0.03	0.65 ± 0.10	0.72 ± 0.03	0.72 ± 0.01
	Leu	1.21 ± 0.09	1.22 ± 0.18	1.24 ± 0.06	1.23 ± 0.01
	Met	0.40 ± 0.04	0.42 ± 0.08	0.40 ± 0.02	0.40 ± 0.02
	Arg	0.85 ± 0.11	0.91 ± 0.19	0.96 ± 0.02	0.93 ± 0.02
	His	0.29 ± 0.04	0.32 ± 0.05	0.34 ± 0.01	0.33 ± 0.01
	Ile	0.68 ± 0.07	0.69 ± 0.09	0.69 ± 0.01	0.69 ± 0.01
TAA		13.97 ± 0.35^b^	14.51 ± 0.20^b^	15.14 ± 0.23^a^	15.04 ± 0.19^a^
EAA/TAA (%)		39.60	38.13	38.58	38.02
DAA/TAA (%)		49.23	48.88	47.68	47.52
SAA/TAA (%)		21.29	22.15	23.57	23.87
BAA/TAA (%)		29.48	28.97	28.75	28.61

Asp is aspartic acid; Ala is alanine; Arg is arginine; Glu is glutamate; Gly is glycine; His is histidine; Ile is isoleucine; Leu is leucine; Lys is lysine; Met is methionine; Phe is phenylalanine; Pro is proline; Ser is serine; Thr is threonine; Tyr is tyrosine; Val is valine; TAA, is total amino acids; EAA, is essential amino acids; DAA, is delicious amino acids; SAA, is sweet amino acids; BAA, is bitter amino acids; and FW, is fresh weight. Lowercase letters indicate the significant difference between the different groups (*P* < 0.05). The type of error bars is standard deviation (SD).

Unlike the amino acids, the fatty acids did not reveal a dominant fatty acid, as the concentrations of all fatty acids measured were below the threshold of 1.00 g/100 g (Table 2). A total of seven fatty acids with significant differences between groups were identified (*P* < 0.05), including hexadecanoic acid (C16:0), cis-9-hexadecane-1-enoic acid (C16:1n7), octadecanoic acid (C18:0), cis-9-octadecenoic acid (C18:1n9c), cis, cis 9,12-octadecadienoic acid (C18:2n6c), cis, cis 11,14-eicosadienoic acid (C20:2n6), and cis-4,7,10,13,16,19-docosahexaenoic acid (C22:6n3). Of these, most fatty acids were the lowest in the GSP-supplemented groups (TL, TM, and TH groups) in comparison to the blank control (CK group), with the exception of C18:1n9c, which was the lowest in the CK group. Therefore, it can be concluded that dietary supplementation with GSP resulted in a decrease in muscle fatty acids.

**TABLE 2:** The muscle fatty acids composition of grass carp after supplemental feeding with procyanidin-rich GSP (n = 3).

Fatty acids (g/100 g)	CK	TL	TM	TH
Hexadecanoic acid (C16:0)	0.112 ± 0.016^a^	0.107 ± 0.015^a^	0.091 ± 0.018^ab^	0.076 ± 0.008^b^
Cis-9-hexadecane-1-enoic acid (C16:1n7)	0.013 ± 0.004^a^	0.010 ± 0.003^ab^	0.009 ± 0.003^ab^	0.005 ± 0.002^b^
Cis-10 heptadecaenoic acid (C17:1n7)	0.010 ± 0.003	0.016 ± 0.007	0.011 ± 0.004	0.008 ± 0.001
Octadecanoic acid (C18:0)	0.051 ± 0.010^ab^	0.061 ± 0.011^a^	0.045 ± 0.012^ab^	0.038 ± 0.006^b^
Cis-9-octadecenoic acid (C18:1n9c)	0.012 ± 0.003^b^	0.051 ± 0.015^a^	0.022 ± 0.018^ab^	0.036 ± 0.025^ab^
Cis, cis 9,12-octadecadienoic acid (C18:2n6c)	0.065 ± 0.013^a^	0.041 ± 0.002^b^	0.054 ± 0.018^ab^	0.065 ± 0.005^a^
Cis, cis, cis-9,12,15-octadecyltriaenoic acid (C18:3n3)	0.005 ± 0.001	-	0.003 ± 0.001	0.003 ± 0.001
Cis, cis 11,14-eicosadienoic acid (C20:2n6)	0.004 ± 0.001^a^	0.001 ± 0.001^b^	0.004 ± 0.001^a^	0.006 ± 0.001^a^
Cis, cis, cis-8,11,14-eicosotrienic acid (C20:3n6)	0.011 ± 0.002	0.010 ± 0.001	0.010 ± 0.002	0.012 ± 0.001
Cis-5,8,11,14-eicosatetraenoic acid (C20:4n6)	0.058 ± 0.011	0.061 ± 0.002	0.055 ± 0.005	0.054 ± 0.004
Cis-5,8,11,14,17-eicosapentaenoic acid (C20:5n3)	0.007 ± 0.001	0.004 ± 0.002	0.005 ± 0.001	-
Docosatetraenoic acid (C22:4n6)	-	-	0.004 ± 0.001	0.003 ± 0.001
Docosapentaenoic acid (C22:5n6)	0.031 ± 0.006	0.032 ± 0.002	0.032 ± 0.006	0.034 ± 0.004
Cis-4,7,10,13,16,19-docosahexaenoic acid (C22:6n3)	0.042 ± 0.004^a^	0.032 ± 0.008^ab^	0.026 ± 0.004^b^	0.022 ± 0.002^b^
TFA	0.423 ± 0.040	0.427 ± 0.053	0.372 ± 0.047	0.361 ± 0.037

TFA, is total fatty acids content; FW, is fresh weight; and “-” represents no detection. Lowercase letters indicate the significant difference between the different groups (*P* < 0.05). The type of error bars is standard deviation (SD).

### 3.7 Muscle safety inspection of grass carp

As shown in Supplementary Table S4, the contents of Pb, Cd, Hg, and As were lower than 0.2, 0.05, 0.05, and 0.1 mg/kg, respectively, which were all below the permissible limits (GB 2762-2022 and GB 2763-2021). Additionally, there were no detectable food pathogenic microbes (*Salmonella* and *Escherichia coli*) and pesticide residues (DDT, HCH, and PCNB) in the muscle. These findings clearly demonstrated that the muscle of grass carp was safe for consumption after supplemental feeding with GSP.

### 3.8 Gut microbiome analysis of grass carp

**FIGURE 5:**
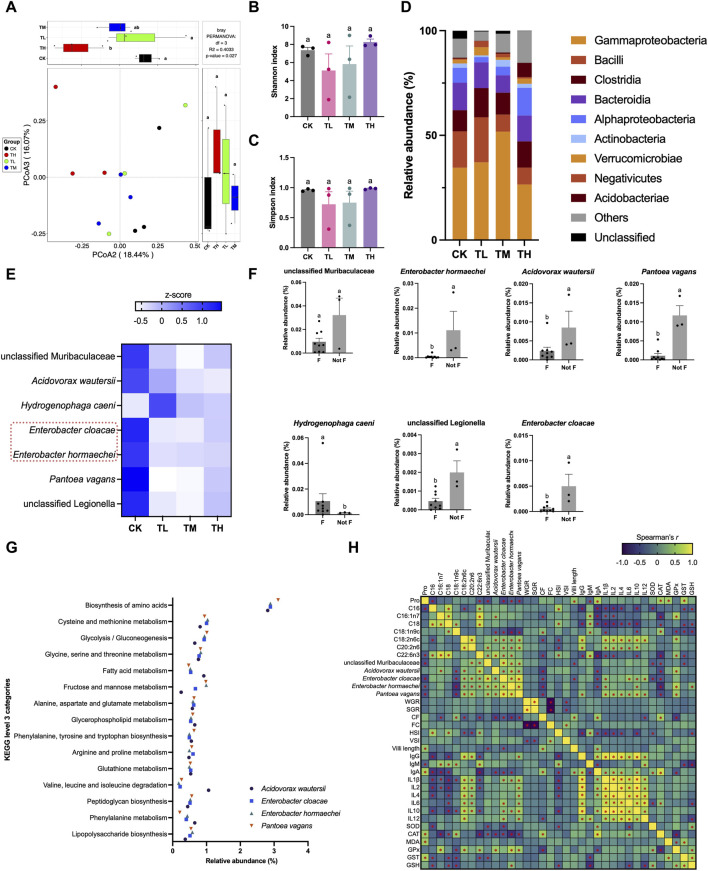
Gut microbiota in grass carp after supplemental feeding with procyanidin-rich GSP (n = 3). **(A)**, PCoA plot showing the difference in gut microbiota between the different groups; **(B,C)**, histograms showing the difference in alpha diversity between the different groups (**B**, Shannon index; **C**, Simpson index) (One-way ANOVA for Shannon and Simpson index, *P* < 0.05); **(D)**, stacked plot showing the relative abundance of gut microbiota at the class level; **(E,F)**, a random forest (RF) test revealed the keystone species in the gut microbiota **(E)** heatmap showing the seven selected keystone species between the different groups; **(F)** the relative abundance of the seven selected keystone species between the blank control (Not F) and the GSP-supplemented groups **(F)** (non-parametric *t*-test, *P* < 0.05); **(G)**, scatter plot showing the relative abundance of the imputed functional profiles related to amino acids and fatty acids in the keystone species; and **(H)**, heatmap showing the Spearman’s correlation between the altered amino acid (Pro) and fatty acids (C16:1n7, C18:0, C18:1n9c, C18:2n6c, C20:2n6, and C22:6n3), the relative abundance of keystone species (unclassified *Legionella*, *Acidovorax wautersii*, *Enterobacter hormaechei*, *Enterobacter cloacae*, and *Pantoea vagans*), the growth parameters (WGR, SGR, CF, FC, HSI, and VSI), the histological changes (villi length), the immunologic indexes (IgG, IgA, IgM, IL-1*β*, IL-2, IL-4, IL-6, IL-10, and IL-12), and the antioxidative indexes in gut (SOD, CAT, MDA, GPx, GST, and GSH). Lowercase letters indicate the significant difference between the different groups (*P* < 0.05). “*” showing significant correlation in Spearman correlation analysis. The type of error bars is standard deviation (SD).

Although no significant differences were identified between the CK group and the GSP-supplemented groups (TL, TM, and TH groups) (*P* > 0.05) based on alpha-diversity (Shannon and Simpson indexes), significant compositional changes in the gut microbiota were observed between groups based on beta-diversity (*P* < 0.05) (Figures 5A–C). Specifically, the class Gammaproteobacteria was the dominant taxon across the four tested groups, exhibiting the highest relative abundance (Figure 5D). Furthermore, class Gammaproteobacteria, Bacilli, Clostridia, Bacteroidia, and Alphaproteobacteria were the top five taxa as their relative abundance constituted the largest proportion among the classes. Seven keystone taxa were selected based on the random forest test (Figure 5D). Of these taxa, the relative abundance of *Enterobacter hormaechei*, *Enterobacter cloacae*, *Acidovorax wautersii*, *Pantoea vagans*, and unclassified *Legionella* significantly decreased in the GSP-supplemented groups (TL, TM, and TH groups) when compared to the blank control (CK group) (*P* < 0.05), whereas the relative abundance of *Hydrogenophaga caeni* significantly increased (*P* < 0.05) (Figures 5E,F). Moreover, *in vitro* inoculation trials with two typical pathogenic bacteria found in the gut (*E. hormaechei* and *E. cloacae*) were conducted, demonstrating excellent antibacterial effects under GSP treatments since the inhibition rates exceeding 95% (Supplementary Table S5). These findings supported the results of *in vivo* gut microbial sequencing. Additionally, the four downregulated keystone species appeared to be involved in 15 KEGG pathways connected to either amino acid or fatty acid metabolism/biosynthesis (Figure 5G). Interestingly, significant correlations were identified between the relative abundance of keystone species and the altered levels of amino acids and fatty acids, wherein *E. hormaechei* and *E. cloacae* exhibited a negative relationship with the contents of Pro and C18:1n9c (*P* < 0.05) and a positive correlation with the contents of C18:2n6c, C20:2n6, and C22:6n3 in muscle (*P* < 0.05) (Figure 5H). Moreover, *A. wautersii* revealed significant correlations with the contents of C16:1n7, C18:1n9c, C18:2n6c, and C22:6n3 (*P* < 0.05), while *P. vagans* exhibited significant correlations with the contents of Pro, C18:1n9c, C18:2n6c, and C20:2n6. These results suggested that dietary supplementation with GSP induced compositional changes in muscle nutrition through gut microbial mediation.

### 3.9 Schematic route of GSP facilitating the growth of grass carp

**FIGURE 6:**
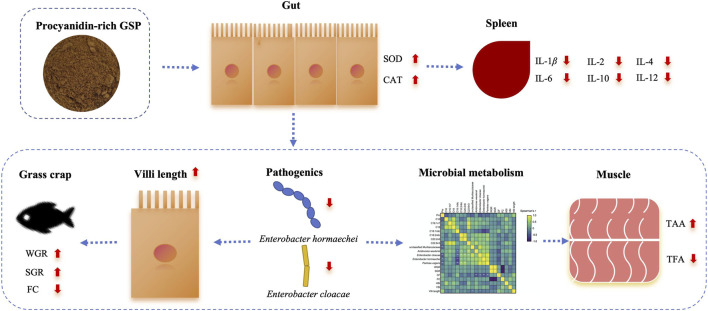
The schematic route of procyanidin-rich GSP facilitating the growth performance of grass carp.

Comprehensive the above-mentioned results, we constructed a schematic route detailing the role of GSP in facilitating the growth of grass carp (Figure 6). In this framework, feeding inclusion GSP positively influenced gut microbiota, inhibited pathogenic *Enterococcus*, and subsequently altered the composition of amino acids and fatty acids via gut microbial mediation. Furthermore, a healthy gut environment facilitated an increase in villi length, which resulted in both growth promotion and a reduction in feed conversion ratio. Additionally, an improvement in antioxidant capacity (particularly in the gut) and a decrease in inflammation levels were observed with daily addition of GSP. These findings suggest that the enhanced growth performance after supplemental feeding with GSP may be attributed to its inflammation-relieving effects.

## 4 Discussion

### 4.1 Dietary supplementation with GSP is responsible for the growth-promoting effect

It is widely acknowledged that incorporating plant-based ingredients into aquaculture feeds enhances nutritional availability and positively affects growth performance (Awad and Awaad, 2017; Hemre et al., 2016). For instance, daily supplementation with extracts that derived from *Ampelopsis grossedentata* (Yang et al., 2024), *Crataegus monogyna* (Tan et al., 2017), and *Panax notoginseng* (Sun et al., 2018) had substantially improved the growth performance of fishes such as *Megalobrama hoffmanni*, *Trachinotus ovatus*, and hybrid grouper (*Epinephelus lanceolatus* ♂ × *Epinephelus fuscoguttatus* ♀), respectively. Grape seeds, recognized as a significant source of bioactive constituents, have been utilized in the poultry and livestock sectors, demonstrating considerable potential for growth-promoting and disease-preventing (Hassan et al., 2019; Huang et al., 2022). However, research regarding the application of grape seeds in aquaculture remains limited. In this study, procyanidin content in the prepared GSP reached above 10%, which was significantly higher than that found in other grape seeds (<2%) globally (Krasteva et al., 2023). Furthermore, literature indicates that the Kyoho grape possessed the highest procyanidin content among 44 evaluated grape varieties (Liao, 2011), supporting the relative high procyanidin content in the prepared GSP. Since daily supplement of procyanidin was growth-promoting (Baron et al., 2024; Ricketts and Ferguson, 2018), we therefore proposed that the prepared GSP was conducive to growth performance elevation in the aquaculture of grass carp. Interestingly, significant increases in the WGR, SGR, and CF of grass carp were noticed after supplemental feeding with the prepared GSP, suggesting that dietary supplementation with GSP indeed elevate the growth performance of grass carp. The growth-promoting effects may be attributed to the ability of GSP to mitigates hepatic disorders (Terzi et al., 2023), regulate gut function (Li et al., 2020), and inhibit oxidative stress (Trapani et al., 2022), which evidenced by the explanations in other fish or fish cells. Furthermore, plant-based ingredients that alter gut histomorphology have been observed, closely linking to fish growth performance (Wang X. et al., 2023). Commonly, an increased villi length was beneficial for nutrient absorption (Chen et al., 2021; Ege et al., 2019). Notably, the villi length of the gut increased in the GSP-supplemented groups compared to the blank control. Consequently, the rate of nutrient absorption and transformation within the gut may be enhanced with daily supplements of GSP, thereby contributing to growth performance elevation in the aquaculture of grass carp. Moreover, a markedly reduction in feed conversion ratio was observed due feeding inclusion GSP, corroborating the findings related to absorption enhancement through villi length elevation. Furthermore, no heavy metals, pesticide residues, or foodborne pathogens were detected in the muscle tissue of grass carp, indicating that dietary supplementation with GSP did not pose any food safety concerns. In conclusion, we affirmed that the prepared GSP could function as a viable feed additive in aquaculture.

### 4.2 GSP supplementation at the low level is suitable for the healthful aquaculture of grass carp

Typically, dietary supplementation with plant-based components at an appropriate dosage is most beneficial for animal production (Zhou et al., 2023). As previously indicated, significantly higher WGR, SGR, and CF were observed with GSP supplementation at low and medium levels in comparison to the blank control. Moreover, a decrease in FC signified an improvement in the feed conversion ratio, and a reduced FC could lead to lower feeding expenses in aquaculture (Elvy et al., 2023). Notably, the FC in the TL group was considerably lower than that in the blank control. Consequently, we speculated that daily addition of GSP at a relative low level was sufficient to stimulate the growth of grass carp. Aside from growth parameters, the physiological and biochemical changes exhibited an elevation in antioxidative capacity with daily supplementation of GSP. Typical antioxidant enzymes, specifically SOD and catalase CAT, participated in the scavenging of free radicals (Naraki et al., 2021) and were upregulated after supplemental feeding with GSP. In addition, an enhanced effects were observed with diet addition of GSP at the low and middle levels. Moreover, MDA, a product of polyunsaturated fatty acids (Tsikas, 2017), can reflect the oxidative injury induced by plant-based ingredients. However, no significant differences in MDA contents were detected between groups. Consequently, it could infer that the daily supplement of GSP at the low and middle levels contributed to the elevation of antioxidant capacity.

Additionally, oxidative injury is commonly associated with an immune response and inflammatory reaction (Iddir et al., 2020). It is noteworthy that all of the selected inflammatory factors decreased if feeding inclusion GSP at the low and middle levels; however, most factors increased if diet addition of GSP at the high level. Numerous evidences had illustrated that feeding inclusion GSP or grape seed extract (GSE) was conducive to inflammation-alleviation in animals, such as rats (Salah et al., 2023), lambs (Mu et al., 2021), and weaned beef calves (Ma et al., 2023), with the dosage of GSP/GSE ranging from 40 to 500 mg/kg. Therefore, dietary supplementation with GSP/GSE at a relative low level contributed to inflammation reduction, which supported our results. However, a elevation in inflammation was triggered if the dosage of GSP/GSE increased. For instance, a pro-inflammatory immune response was observed in broiler chickens with daily addition of GSE at the high level (1,000 mg/kg–4,000 mg/kg) (Huerta et al., 2022). In line with broiler chicken, the inflammatory reaction was stimulated in grass carp with the dosage of GSP reached to 1,000 mg/kg. As a result, it might be a common phenomenon that feeding inclusion GSP at the relative high level would cause inflammatory response and the toxicity of GSP might be dosage-dependent (Lluís et al., 2011). Literature demonstrated that an enhanced antioxidant capacity and a suppression of inflammation could improve the growth performance of fish (Pan et al., 2022). Therefore, it is posited that the growth-promoting effect of GSP on grass carp was closely associated with the elevated antioxidative processes as well as the reduced of inflammation. Considering these aspects, although dietary supplementation with GSP at the low and middle levels displayed similar effects, the low dosage was one-fifth of the middle dosage. Consequently, it was concluded that dietary supplementation of GSP at the relative low level was appropriate for the healthful aquaculture of grass carp.

### 4.3 A healthy gut microbiota is created with daily supplementation of GSP

Previous studies had demonstrated that dietary supplementation with plant-based ingredients significantly influenced the composition of gut microbiota, such as the *Eucommia ulmoides* (Peng et al., 2022) and *Ampelopsis grossedentata* extracts (Yang et al., 2024). A critical mechanism involvement with the alterations in gut microbiota was to recruit probiotics as well as inhibit pathogenic bacteria (Wu et al., 2024). In accordance with previous findings, the pathogenic bacteria *E. hormaechei* and *E. cloacae* in the gut were effectively suppressed after supplemental feeding with GSP when compared to the blank control, a phenomenon substantiated by the *in vitro* inoculation trials where the inhibition rates of GSP against these two pathogenic bacteria exceeded 95%. Furthermore, numerous studies had indicated that gut microbiota was closely linked to amino acid and fatty acid metabolism, thereby influencing the nutritional quality of muscle in animals through gut microbiota mediation (Wang X. et al., 2023). In the current investigation, alterations in amino acids (notable increase in Pro) and fatty acids (notable decreases in C16:0, C16:1n7, C18:0, C18:1n9c, C20:2n6, and C22:6n3) were observed due to feeding inclusion of GSP. Moreover, four keystone species (*E. hormaechei*, *E. cloacae*, *A. wautersii*, and *P. vagans*) were identified and displayed significant correlations with these altered amino acid and fatty acids. Evidence had revealed that the genus *Enterobacter* was closely associated with the fatty acid profiles, e.g., *E. cloacae* was involved in the metabolism of C18:0 (Gu et al., 2022) and Pro (Wang P. et al., 2023). Additionally, *A. wautersii* had been shown to regulate the metabolism of C16:0 and C18:0 (Chen et al., 2024). Consequently, it was concluded that the compositional changes in muscle nutrition of grass carp was associated with diet addition of GSP via gut microbiota mediation. Besides, a healthy gut microenvironment concurrently promoted the changes in gut histomorphology (Yan et al., 2023). As previously noted, the villi length of gut increased with daily supplementation of GSP in comparison to the blank control, demonstrating a significant negative correlation with *E. cloacae* and the unclassified Muribaculaceae (*P* < 0.05). Based on these results, it could speculate that dietary supplementation with GSP fostered a healthy gut by inhibiting pathogenic bacteria, which potentially participated in muscle nutrition alteration. Considering every aspects, the elevation in the growth performance of grass carp as dietary supplementation with GSP was attributed to the following factors: (I) an enhanced antioxidant capacity coupled with a reduction in inflammation levels; (II) the promotion of a healthy gut characterized by a decrease in pathogenic organisms and an increase in villi length; and (III) alterations in muscle nutritional composition mediated by gut microbiota.

Although a preliminary result has been obtained, the accurate conclusion should be verified by a scale-up experiment due to the limitations of small sample size, short feeding period, and the juvenile grass carp used in the present study, which would guide our further research.

## 5 Conclusion

This study elucidated the growth-promoting effects of GSP in aquaculture by using grass carp as experimental animal. Firstly, the growth parameters, including WGR, SGR, and CF, were elevated in the GSP-supplemented groups, indicating that feeding inclusion GSP was growth-promoting for grass carp. Secondly, an upregulation of SOD and CAT, alongside a downregulation of inflammatory indices (IL-1*β*, IL-2, IL-4, IL-6, IL-10, and IL-12), was noted as dietary supplementation GSP at the relative low level, suggesting that an appropriate dosage elevated antioxidant activity and alleviated inflammation. Thirdly, the prepared GSP facilitated the villi length of gut and fostered a healthy gut environment by inhibiting pathogenic bacteria, thereby enhanced nutrient absorption and transformation. Finally, variations in gut microbiota played a significant role in amino acid and fatty acid metabolism, subsequently influencing the nutritional quality of muscle. Our findings concluded that incorporation of GSP in basal feeds was advantageous for promoting the growth performance of grass carp and laid a scientific foundation for its application for serving as feed additive in aquaculture.

## Data Availability

The raw data supporting the conclusions of this article will be made available by the authors, without undue reservation.
