# Supplementary material for: Effects of dietary supplementation with grape seed powders on growth performance and muscle nutrition of grass carp (Ctenopharyngodon idella) by gut microbiota mediation
Source: Front Physiol. 2025 Dec 12;16:1683389. doi: 10.3389/fphys.2025.1683389 (PMC12740752; doi:10.3389/fphys.2025.1683389)
Supplement: Supplementary file 1 [file DataSheet1.docx]

**Supplementary Material**

**Effects of dietary supplementation with procyanidin-rich grape seed powders on growth performance and muscle nutrition of grass carp (*Ctenopharyngodon idella*) by gut microbiota mediation**

Shuaipeng Ma^a^, Qing Liu^c^, Qianqian Chen^a^, Songqing Nie^a^, Yulin Zhang^a^, Gang Wu^a,*^, and Xuesong Wang^b,*^

^a^ Guangdong Eco-engineering Polytechnic, Guangzhou 510520, China;

^b^ Institute of Medical Plant Physiology and Ecology, School of Pharmaceutical Sciences, Guangzhou University of Chinese Medicine, Guangzhou 510006, China;

^c^ Guangzhou Experimental Station of Chinese Academy of Tropical Agricultural Sciences, Guangzhou 510140, China;

* **Corresponding Author:**

Gang Wu Email: 1429693907@qq.com;

Xuesong Wang Email: [wlwxs57813@gzucm.edu.cn;](mailto:xhtxr0901@163.com;)

Tel: +86 020 39358253

Fax number: +86 020 39358253


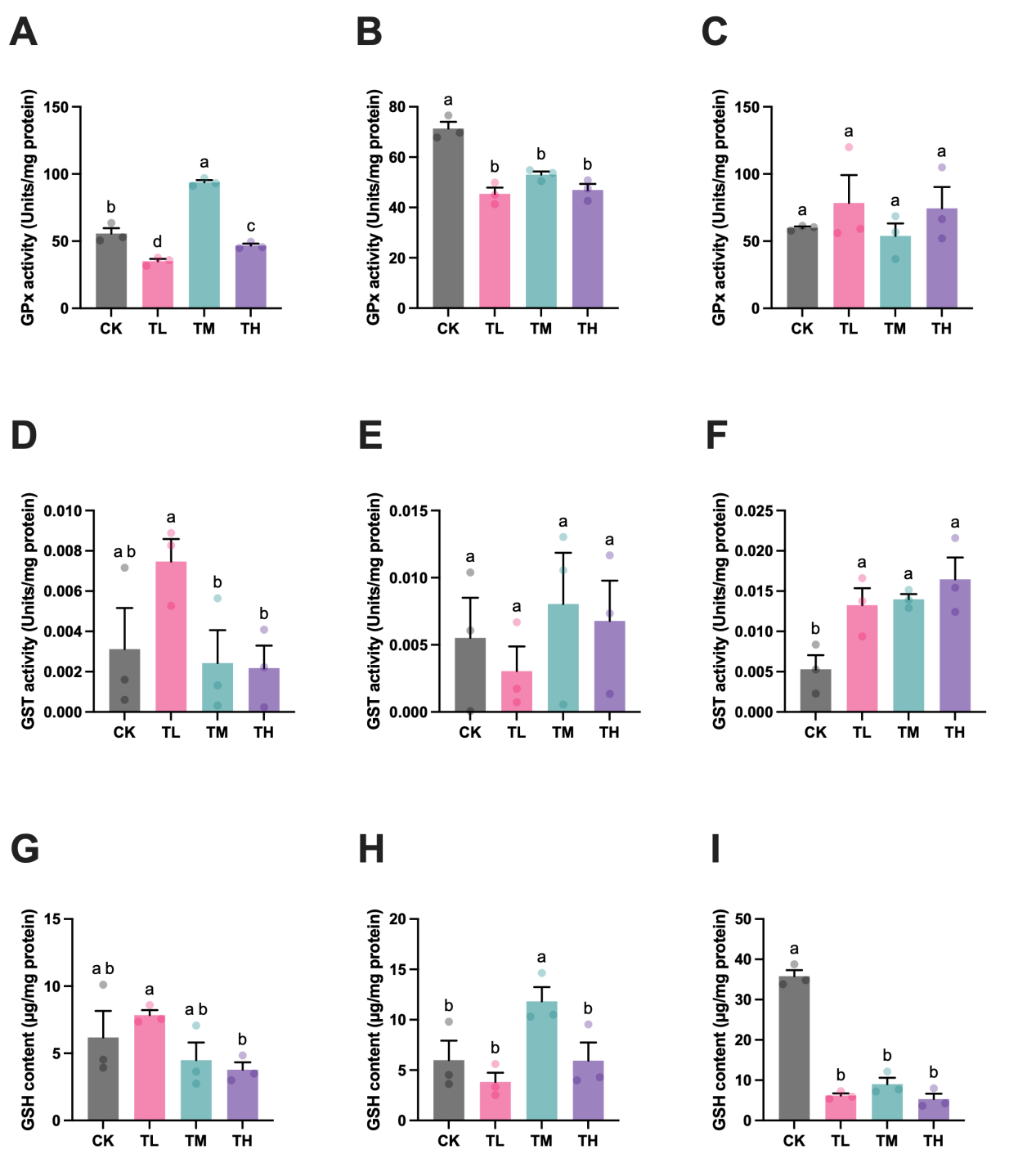


**Fig. S1**. The activities/content of detoxification enzymes/component in tissues of grass carp after supplemental feeding with procyanidin-rich GSP (n = 10). (**A**, **D**, and **G**), gill; (**B**, **E**, and **H**), gut; and (**C**, **F**, and **I**), muscle. Lowercase letters indicate the significant difference between the different groups (*P* < 0.05). The type of error bars is standard deviation (SD).

**Table S1**. The water parameters during the acclimatization period. The type of error bars is standard deviation (SD).

| Item | Concentration |
| --- | --- |
| Dissolved oxygen (DO) | 8.2 ± 0.3 mg/L |
| pH | 7.4 ± 0.2 |
| Water hardness (expressed as CaCO_3_) | 34.8 ± 0.5 mg/L |
| Salinity | 0.10 ± 0.03‰ |
| Nitrate nitrogen (NO₃-N) | 1.4 ± 0.1 mg/L |
| Temperature | 25 ± 0.5℃ |

**Table S2.** The composition of the commercial basal feed pellets.

| Item | Content (%) |
| --- | --- |
| Protein | 28–40 |
| Crude fat | ≥ 3 |
| Crude fibre | ≤ 10 |
| Crude ash | ≤ 12 |
| Moisture | ≤ 10 |
| Total phosphorus | ≥ 1.0 |
| Lysine | ≥ 1.65 |
| Ca | 0.5–2.0 |
| NaCl | 0.3–1.2 |

**Table S3.** The material composition of the prepared GSP. The type of error bars is standard deviation (SD).

| Category | Index | Content |
| --- | --- | --- |
| Bioactive constituents | Procyanidine (g/100 g) | 10.40 ± 0.12 |
|  | Polyphenol (g/100 g) | 25.80 ± 2.13 |
|  | Flavonoid (g/100 g) | 3.56 ± 0.27 |
|  | Polysaccharide (g/100 g) | 0.39 ± 0.02 |
| Heavy metals | Pb (mg/kg) | 0.12 ± 0.01 |
|  | Cd (mg/kg) | ND |
|  | Hg (mg/kg) | ND |
|  | As (mg/kg) | ND |
| Pesticide residues | Dichlorodiphenyl Trichloroethane (mg/kg) | ND |
|  | Hexachlorocyclohexane (mg/kg) | ND |
|  | Quintozene (mg/kg) | ND |
| Microbial contents | Salmonella (25 g) | ND |
|  | *Staphylococcus aureus* (25 g) | ND |
|  | Aerobic bacterial count (CFU/g) | < 10 |
|  | Moulds and Yeasts (CFU/g) | < 10 |
|  | Coliforms (MPN/g) | < 0.3 |

**Table S4.** Muscle safety inspection of grass carp (n = 3). DDT is dichlorodiphenyl trichloroethane; HCH is hexachlorocyclohexane; and PCNB is pentachloronitrobenzene.

| Category | Items | CK | TL | TM | TH |
| --- | --- | --- | --- | --- | --- |
| Heavy metals | Pb (mg/kg) | < 0.2 | < 0.2 | < 0.2 | < 0.2 |
|  | Cd (mg/kg) | < 0.05 | < 0.05 | < 0.05 | < 0.05 |
|  | Hg (mg/kg) | < 0.05 | < 0.05 | < 0.05 | < 0.05 |
|  | As (mg/kg) | < 0.1 | < 0.1 | < 0.1 | < 0.1 |
| Pesticide residues | DDT (mg/kg) | - | - | - | - |
|  | HCH (mg/kg) | - | - | - | - |
|  | PCNB (mg/kg) | - | - | - | - |
| Food pathogenic microbes | Salmonella (CFU/mL) | - | - | - | - |
|  | *Escherichia coli* (CFU/mL) | - | - | - | - |

**Table S5.** *In vitro* inhibition rates of procyanidin-rich GSP against gut pathogenic *Enterobacter cloacae* and *Enterobacter hormaechei*. The type of error bars is standard deviation (SD).

| Bacteria | Microbial concentration  (CFU/mL) | Inhibition rate (%) | |
| --- | --- | --- | --- |
|  |  | 5 (mg/mL) | 25 (mg/mL) |
| *Enterobacter cloacae* | 1×10^3^ | 95.32 ± 0.34 | 98.25 ± 0.28 |
|  | 1×10^4^ | 95.85 ± 0.51 | 98.54 ± 0.43 |
| *Enterobacter hormaechei* | 1×10^3^ | 97.86 ± 0.19 | 99.58 ± 0.31 |
|  | 1×10^4^ | 98.70 ± 0.27 | 98.36 ± 0.54 |
